# Supplementary material for: Organic magnetic nanoparticles catalyze CO2 capture in hydrogen-bonded nanocages via water-driven crystallization
Source: Nat Commun. 2025 Apr 18;16:3702. doi: 10.1038/s41467-025-58734-1 (PMC12008246; doi:10.1038/s41467-025-58734-1)
Supplement: Supplementary file 1 — Supplementary Information [file 41467_2025_58734_MOESM1_ESM.pdf]

Supplementary Information for

Organic magnetic nanoparticles catalyze CO_2 capture in hydrogen-bonded nanocages via water-driven crystallization

Tian Wang[1,2], Aliakbar Hassanpouryouzband[3]*, Mengge Fan[4], Chalachew Mebrahtu[2], Lunxiang Zhang[1]*, Yongchen Song[1]*

[1] *Key Laboratory of Ocean Energy Utilization and Energy Conservation of the Ministry of Education, School of Energy and Power Engineering, Dalian University of Technology, Dalian 116024, China*

[2] *Chair of Heterogeneous Catalysis and Technical Chemistry, Institute for Technical and Macromolecular Chemistry (ITMC), RWTH Aachen University, Worringerweg 2, 52074 Aachen, Germany*

[3] *School of Geosciences, University of Edinburgh, Grant Institute, West Main Road, Edinburgh EH9 3FE, UK*

[4] *School of Environmental Science and Engineering, Guangdong Provincial Key Laboratory of Environmental Pollution Control and Remediation Technology, Sun Yat-sen University, Guangzhou, 510275, China*

* Corresponding Author: Hssnpr@ed.ac.uk (A.H.); lunxiangzhang@dlut.edu.cn (L.Z.); songyc@dlut.edu.cn (Y.S.)

This Supplementary Information includes:

Supplementary Notes 1 to 6

Supplementary Figures 1 to 18

Supplementary Tables 1 to 7

1 Supplementary Notes

Supplementary Note 1: MD simulations

MD calculations were performed by Gromacs 2024.3, and visual analysis was conducted with VMD 1.9.3. The TIP4P/ice model was used for water molecules, and the EPM2 force field was applied to CO_2 molecules. The total simulation time was 2 μs, with a time step of 2 fs. A Nose-Hoover thermostat was used to regulate the temperature to 260 K, and a Parrinello-Rahman barostat was employed to control the pressure to 50 MPa. The temperature and pressure conditions used for the simulation were different from the experimental conditions because the actual nucleation and growth of macroscopic CO_2@Water crystals typically require several hours to even days, and co-crystallization of CO_2 and water molecules is nearly impossible in such conditions under the influence of high Gibbs free-energy barrier between solid and liquid phases. Therefore, ultra-low temperature and ultra-high pressure conditions were adopted to accelerate the nucleation of CO_2@Water crystals to elaborate on the characteristics and formation mechanism of hydrogen-bonded water cages. Prior to MD simulation, energy minimization was performed to eliminate unreasonable atomic contacts. Long-range electrostatic interactions with a cutoff radius of 1 nm were determined using the Particle Mesh Ewald (PME) method. Periodic boundary conditions were applied in all directions. The model contained 2944 water molecules and 512 CO_2 molecules, with an initial model size of 4.7 nm × 4.7 nm × 4.7 nm.

Supplementary Note 2: Hydrogen-bonded arrangement of water molecules via Raman spectra measurement

Raman spectroscopy provides a convenient method of qualitative and quantitative analysis for measuring water structure in single/complex environments. In this work, Raman spectroscopy was utilized to probe the hydrogen bonding network structure of water molecules in pure water and MFNs-0.5 dispersion. The Raman spectra from 2700 cm^{-1} to 3900 cm^{-1} were fitting to five Gaussian peaks locating at 3050 cm^{-1}, 3228 cm^{-1}, 3413 cm^{-1}, 3540 cm^{-1}, and 3635 cm^{-1}, respectively.[1] The peaks at 3050 cm^{-1} and 3228 cm^{-1} are associated with the ordered tetrahedral hydrogen-bonded arrangement (ohb) of water molecules, and the peaks at 3413 cm^{-1}, 3540 cm^{-1}, and 3635 cm^{-1} correspond to the disordered hydrogen-bonded arrangement (dhb) of water molecules.[2] The ordering of hydrogen-bonded arrangement of water molecules was quantified by the integral strength ratio of ohb water molecules to dhb water molecules (I_{ohb}/I_{dhb}).

Supplementary Note 3: In-situ DSC experiment

A high pressure differential scanning calorimeter (HP μ-DSC7 Evo, Setaram) was employed to in-situ determine the effect of MFNs on the kinetic performance of CO_2 capture by monitoring the heat flow changes. The reaction was carried out at a constant pressure of 4 MPa with an initial temperature of 288 K. The temperature was reduced to the experimental temperature (275 K) at a rate of 0.5 K/min and then maintained constant, with a fluid mass of 30 mg for a total reaction time of 10 h.

Supplementary Note 4: In-situ Raman experiment

A LabRAM HR evolution confocal Raman spectrometer equipped with an 1800 grooves/mm grating was employed to in-situ determine the growth kinetics of hydrogen-bonded water cages. The laser excitation wavelength was 532 nm, output power was 100 mW, acquisition range was 1200 to 1500 cm^{-1}, and the spectra was acquired three times with exposure time of 60 s. A capillary optical cell with an inner diameter of 300 μm was placed on a high-pressure cooling table as the reactor. The liquid amount for reaction was 1 μL, and the experimental conditions were kept constant at 4 MPa and 275 K. CO_2 gas signal was initially scanned as a reference, followed by microscopic observation of liquid state in the capillary cell. And once CO_2@Water crystals were detected, the position was immediately focused and Raman spectra were continuously acquired at 5 min intervals, with a reaction time of 45 min. The two peaks at 1286.5 and 1389.3 cm^{-1} were corresponded to CO_2 gas, the signals of CO_2 trapped by hydrogen-bonded water cages were shifted to 1276.3 and 1383.8 cm^{-1} in pure water, respectively. And the peaks of CO_2 captured in the MFNs dispersion located at 1275.9 and 1384.5 cm^{-1}, respectively, which were almost the same as that of pure water, indicating the hydrogen-bonded water cages were not destroyed by MFNs.

Supplementary Note 5: Avrami model

The gas uptake/water conversion curves were fitted to the Avrami model, which could be used to describe the crystallization kinetics of CO_2@Water clathrate under isothermal and isochoric conditions. The Avrami model was constructed according to the assumption of spatially random nucleation, as shown in equation (5) and (6):[3, 4]

$$\alpha = 1 - \exp(-kt^n) \tag{5}$$

$$\ln[-\ln(1-\alpha)] = n\ln t + \ln k \tag{6}$$

Where, α presents the water to clathrate conversion at time t which begins from the nucleation point. n is the Avrami exponent which reveals the mechanism of crystal nucleation and growth. The value of n is composed of two parameters: n_d and n_n. In which, n_d presents the dimensionality of the crystal growth with the value of 1, 2 or 3. However, n_d decreases to almost half with the increase of mass transfer resistance. The value of n_n equals to 0 or 1 based on the type of nucleation, where 0 corresponds to instantaneous or heterogeneous nucleation and 1 to sporadic or homogenous nucleation.[5] However, in many cases, the nucleation may be in between completely instantaneous or completely sporadic, which can lead to non-integer value of n. k presents the rate constant of crystal growth.

Supplementary Note 6: VB-MC Assessment of CO_2 Capture Capacity

The volume-balance method is more accurate than the classic gas-uptake method as it takes into account the volume variation of each phase i (i = Gas, Water and Clathrate) during CO_2@Water clathrate formation (Eq. (1)).

$$V_t^{\mathrm{G}} = V_{\mathrm{R}} - V_t^{\mathrm{W}} - V_t^{\mathrm{C}} = V_{\mathrm{R}} - n_t^{\mathrm{W}} \times v_t^{\mathrm{W}} - n_t^{\mathrm{C}} \times v_t^{\mathrm{C}} \tag{1}$$

where, V_{R} (cm^3) is the total effective volume inside the reactor; n presents the number of moles of CO_2, and v_t^i (cm^3/mol) is the mole volume of the phase i at time t (min). v^{W} (cm^3/mol)is the molar volume of water (18.0 cm^3/mol) ; v^{C} (cm^3/mol) is the molar volume of CO_2@Water calculated by the density of CO_2 hydrate (1.1 g/cm^3).[6]

$$n_t^{\mathrm{W}} = n_{t_0}^{\mathrm{W}} - N_h \times n_t^{\mathrm{C}} \tag{2}$$

where, $n_{t_0}^{\mathrm{W}}$ (g/mol) is the molar amount of initial water injected into the reactor. N_h is the hydration number which was calculated to 6.82 for CO_2@Water clathrate.[7]

The amount of gas consumption (n_t^{G}, mol) was calculated by Eq. (3):

$$n_t = \frac{P_{t_0} V_{t_0}^{\mathrm{G}}}{Z_{t_0} R T_{t_0}^{\mathrm{G}}} - \frac{P_t V_t^{\mathrm{G}}}{Z_t R T_{t_0}^{\mathrm{G}}} \tag{3}$$

where P_t (Pa), V_t (m^3), Z_t and T_t (K) are the pressure, volume, gas compression constant and temperature of gas phase in the reactor at time *t* (min), respectively. t_0 (min) presents the initial time of the experimental procedure. The gas compression constant *Z* was calculated by the Benedict–Webb–Rubin–Starling (BWRS) equation of state, and *R* is the universal gas constant.

The CO_2 storage capacity (*C*-v/v) was calculated by Eq. (4):

$$C = \frac{n_t v^{\mathrm{G}} v^{\mathrm{W}}}{V_{t_0}^{\mathrm{W}} (v^{\mathrm{W}} + \Delta v)} \tag{4}$$

where ν^{G} (cm^3/mol) and ν^{W} (cm^3/mol) are the molar volumes of gas and water, respectively, and $V_{t_0}^{\mathrm{W}}$ (cm^3) is the initial volume of the solution. $\Delta\nu$ (cm^3/mol) is the molar volume difference between water and CO_2@Water clathrate and was calculated to be 4.62 cm^3/mol.[8] Note that the unconverted water was not eliminated in the calculation making the obtained normalized CO_2 storage capacity lower than that actually captured in solid clathrates.

2 Supplementary Figures

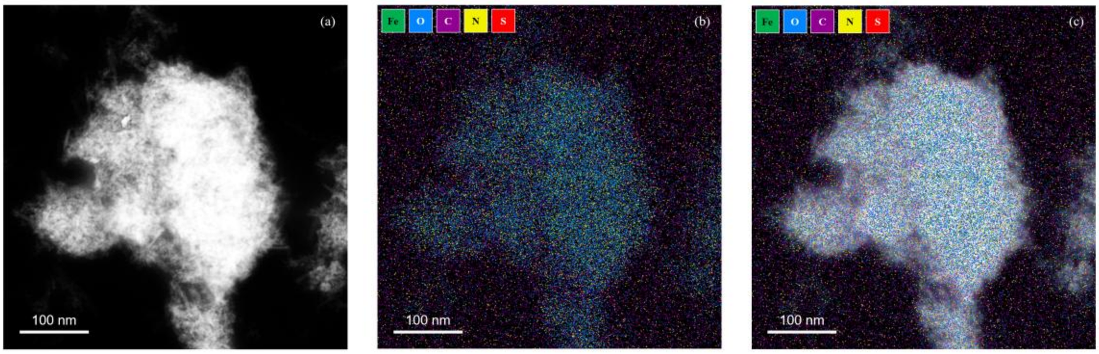

Supplementary Fig. 1. TEM image and the overlap of elemental mappings of Fe, O, C, N, and S for MFNs. (a) TEM image of MFNs. **(b)** Overlap of elemental mappings of Fe, O, C, N, and S, and **(c)** TEM images with all elemental mappings.

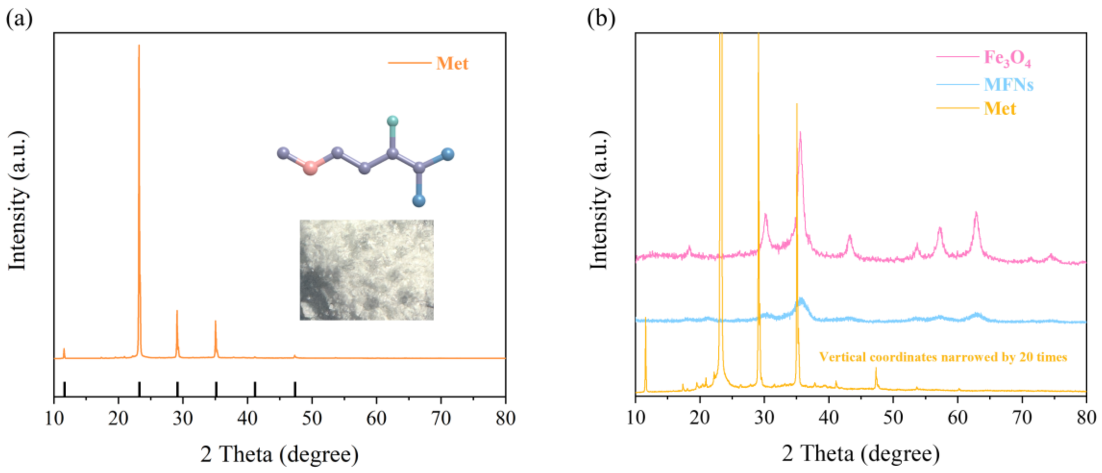

Supplementary Fig. 2. XRD pattern of Methionine (Met) and the comparison of XRD patterns of Fe_3O_4, Met and MFNs. (a) XRD pattern of pure Met. The inset shows a schematic and macroscopic view of Met molecular structure. **(b)** XRD patterns of Fe_3O_4, MFNs, and Met. The signal value of Met is scaled down by a factor of 20 to highlight the remaining weak diffraction peaks. Source data are provided as a Source Data file.

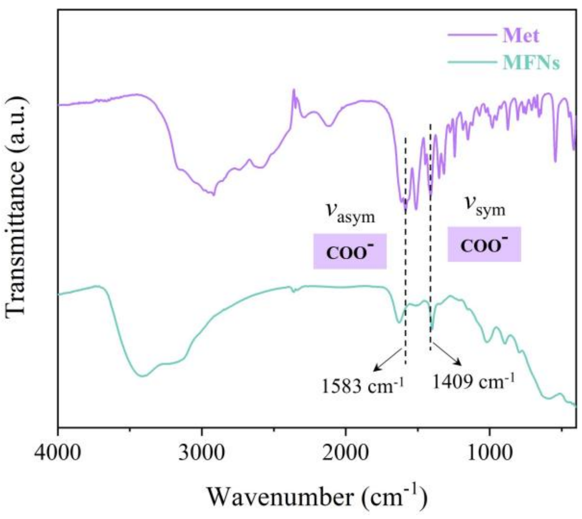

Supplementary Fig. 3. FTIR spectra of Met and MFNs. Source data are provided as a Source Data file.

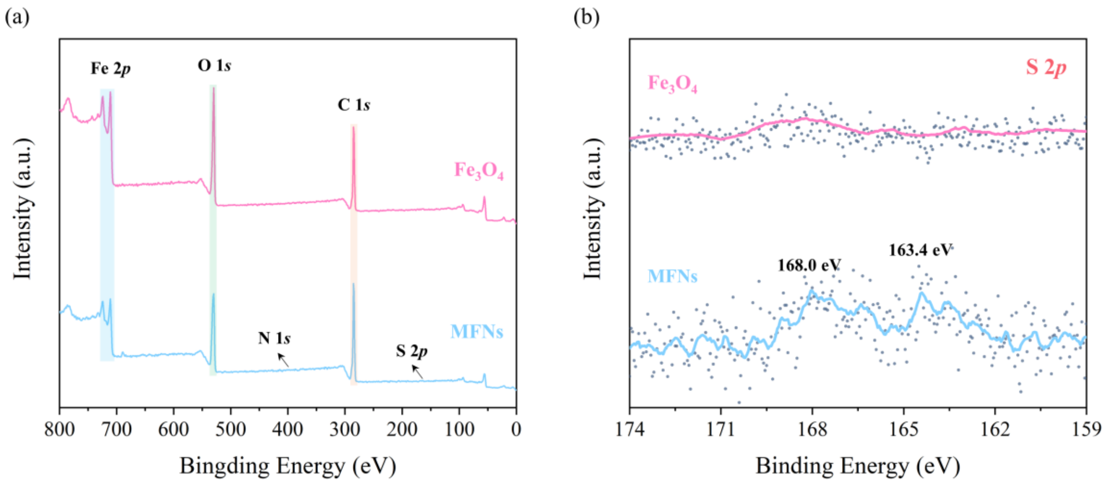

Supplementary Fig. 4. Comparison of XPS survey spectra and high-resolution XPS spectra of S 2*p* of Fe_3O_4 and MFNs. (a) XPS survey spectra of Fe_3O_4 nanoparticles and MFNs. **(b)** High-resolution XPS spectra of S 2*p* of Fe_3O_4 and MFNs. Source data are provided as a Source Data file.

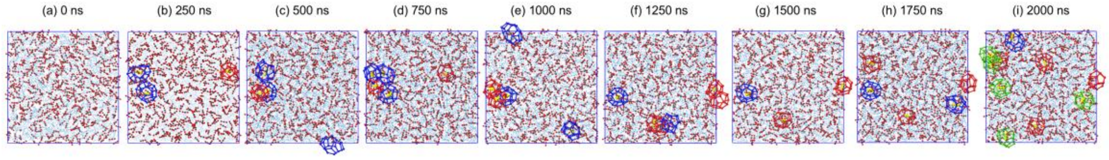

Supplementary Fig. 5. Snapshots of MD simulations for CO_2 capture in hydrogen-bonded water cages within 2000 ns at 250 ns intervals.

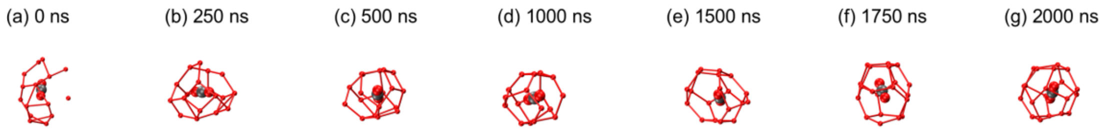

Supplementary Fig. 6. Structure of hydrogen-bonded water cages within 0.5 nm surrounding a CO_2 molecule involved in CO_2@Water crystal nucleation at different simulation moments. CO_2 molecule is represented as a ball-and-stick model consisting of black and red colors, water molecules are represented by small red balls, and hydrogen bonds are represented by red sticks.

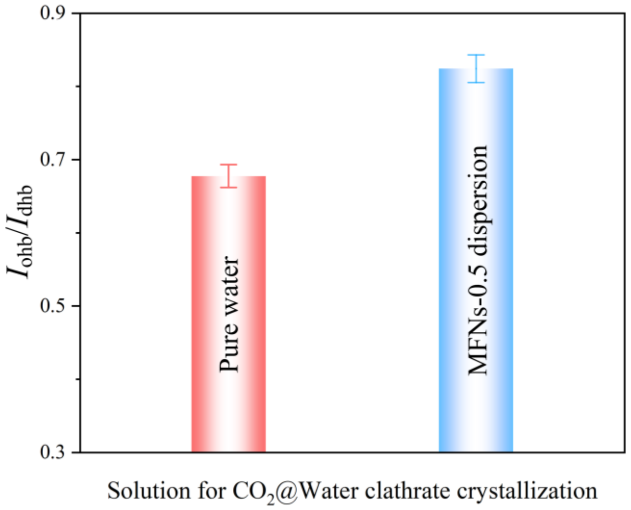

Supplementary Fig. 7. I_{ohb}/I_{dhb} of pure water and MFNs dispersion. The error bars are determined from the standard deviation of three independent measurements. Source data are provided as a Source Data file.

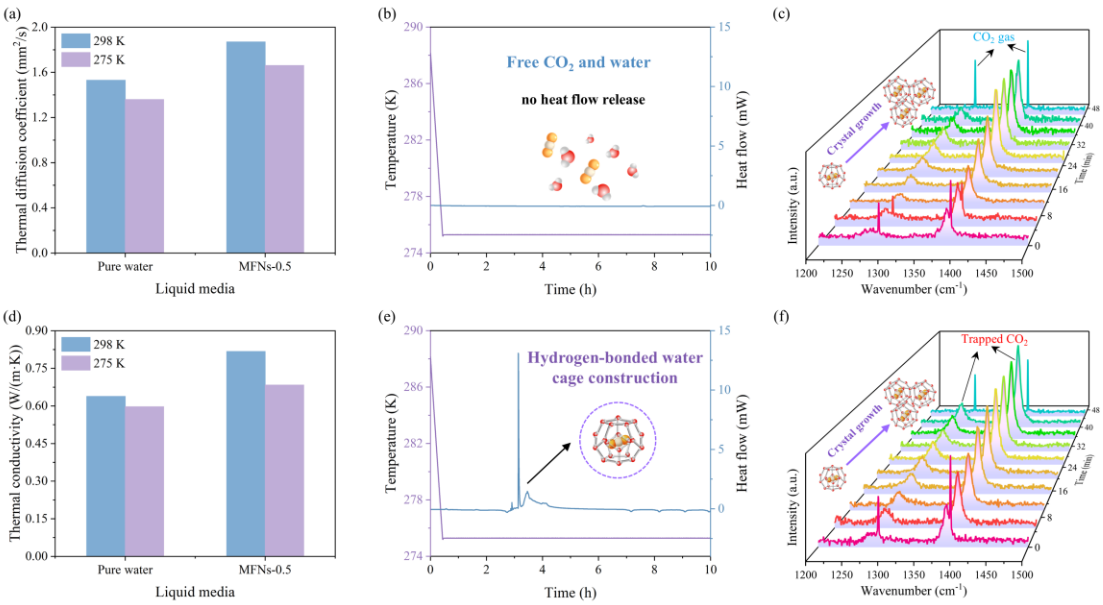

Supplementary Fig. 8. Mechanism analysis of the promotion of MFNs for CO_2 capture based on hydrogen-bonded water cages. Thermal diffusion coefficients **(a)** and thermal conductivities **(d)** of pure water and 0.5 wt% MFNs dispersion at ambient temperature and experimental temperature, respectively. Temperature profiles and heat flow changes of pure water **(b)** and 0.5 wt% MFNs dispersion **(e)** monitored by in-situ DSC, respectively. In-situ time-varying Raman spectra during CO_2 trapping in hydrogen-bonded water cages of pure water **(c)** and 0.5 wt% MFNs dispersion **(f)**, respectively. Source data are provided as a Source Data file.

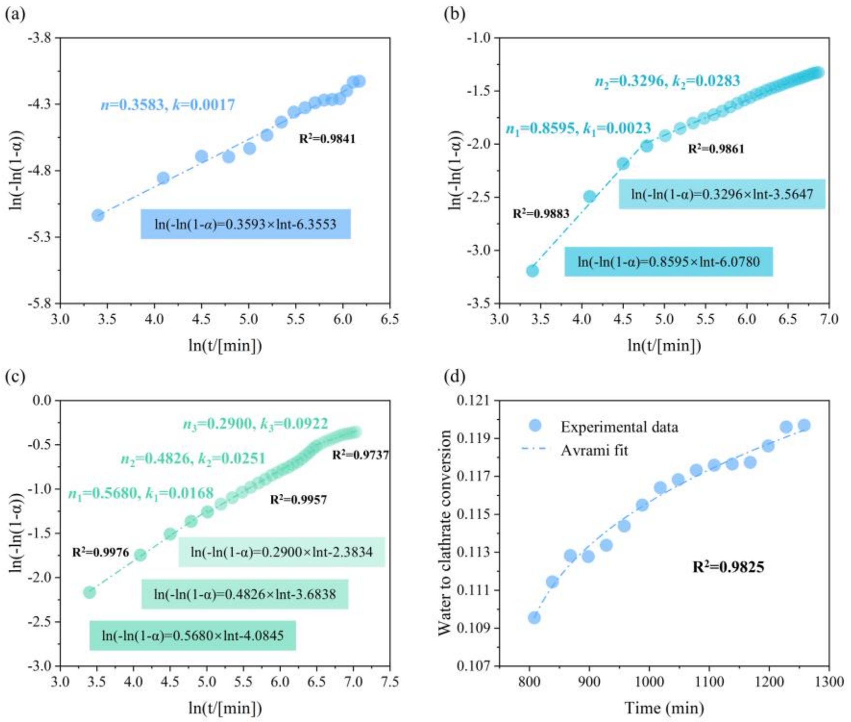

Supplementary Fig. 9. Avrami model fit to CO_2@Water clathrate formation in 0-0.1 wt% MFNs dispersion. The Avrami plot for CO_2@Water clathrate formation in **(a)** pure water; **(b)** MFNs-0.05; **(c)** MFNs-0.1. **(d)** Comparison of measured data and model prediction of water to clathrate conversion in pure water. Source data are provided as a Source Data file.

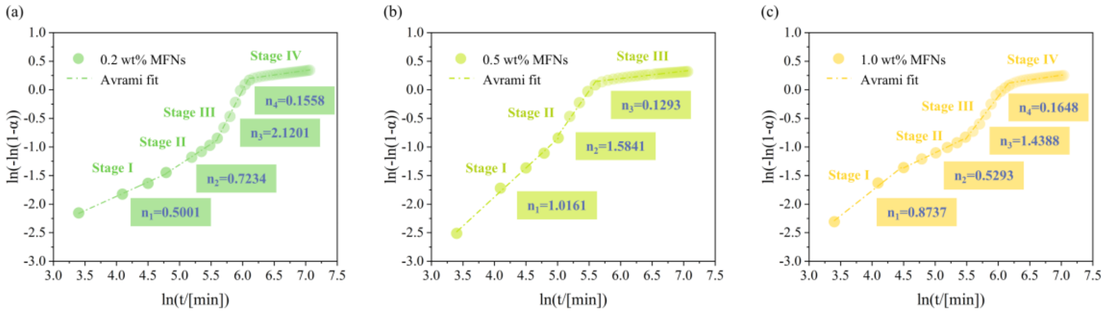

Supplementary Fig. 10. Avrami model fit to CO_2@Water clathrate growth in 0.2-1.0 wt% MFNs dispersion. Avrami plot of multiple stages during CO_2@Water clathrate growth in **(a)** 0.2 wt% MFNs dispersion, **(b)** 0.5 wt% MFNs dispersion, and **(c)** 1.0 wt% MFNs dispersion. Source data are provided as a Source Data file.

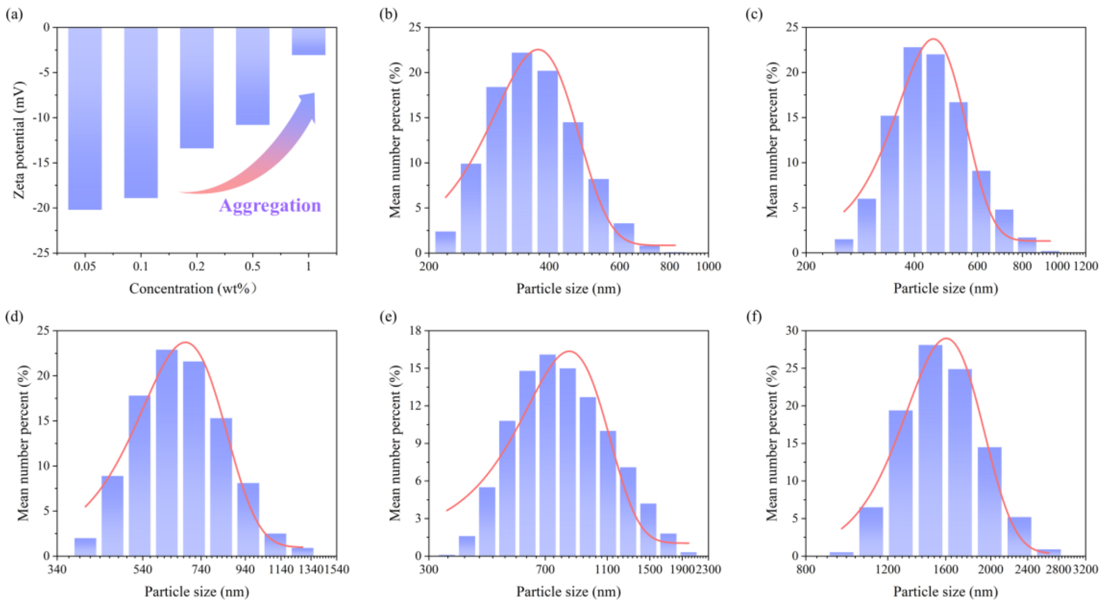

Supplementary Fig. 11. The dependence of MFNs stability on the concentration. (a) Zeta potential of MFNs at different concentrations. **(b)-(f)** Hydrodynamic diameter of MFNs at 0.05, 0.1, 0.2, 0.5, and 1.0 wt% concentrations, respectively. Source data are provided as a Source Data file.

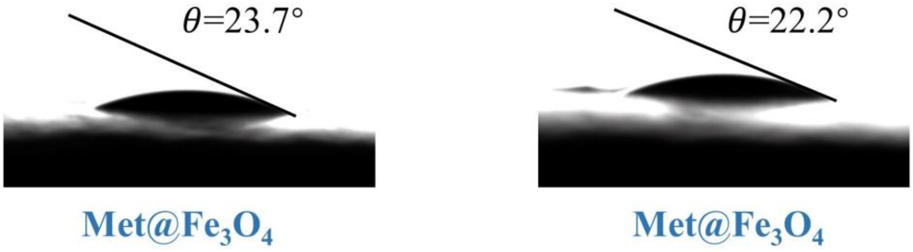

Supplementary Fig. 12. Water contact angle of Met@Fe_3O_4. Met@Fe_3O_4 powder was pressed into flat flakes with a press before testing.

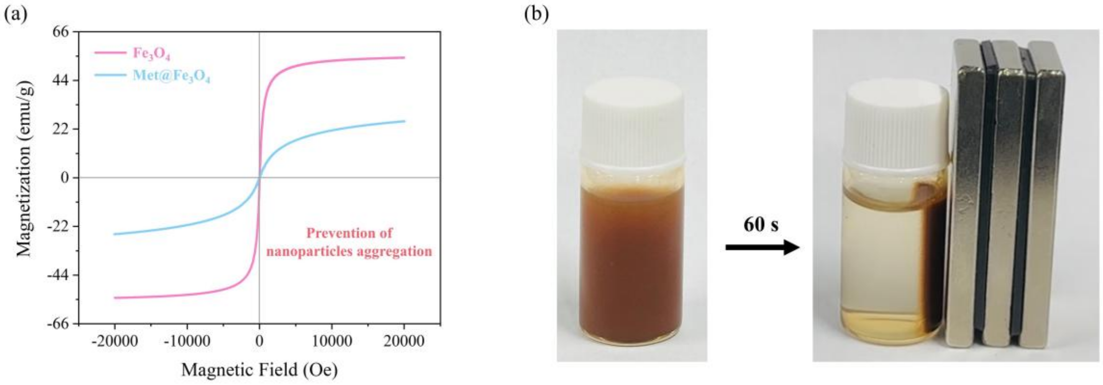

Supplementary Fig. 13. Magnetic properties of MFNs and their recovery performance by magnet. (a) Magnetization curves of the as-prepared MFNs and bare Fe_3O_4 nanoparticles. Source data are provided as a Source Data file. **(b)** Recovery performance of the MFNs samples with a magnet.

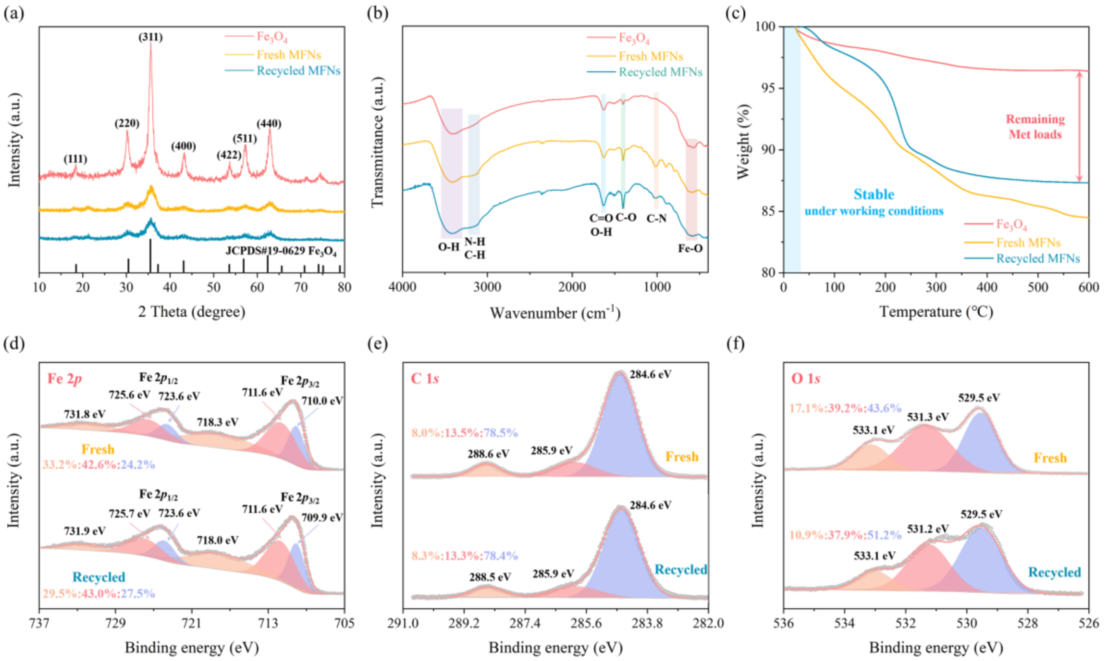

Supplementary Fig. 14. Comparison of physicochemical properties of MFNs before and after recycling test. (a) XRD patterns. **(b)** FT-IR spectra. **(c)** TG curves. **(d-f)** High-resolution XPS spectra of Fe 2*p* **(d)**, C 1*s* **(e)**, and O 1*s* **(f)** for fresh and recycled MFNs. Source data are provided as a Source Data file.

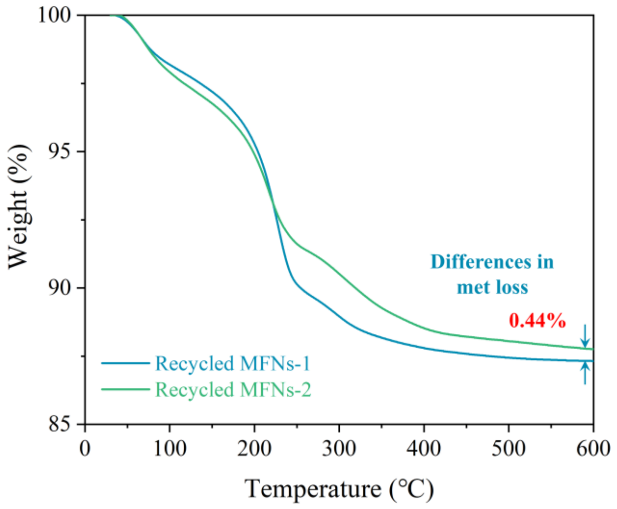

Supplementary Fig. 15. Comparison of TG curves of two MFNs tested over 17 cycles. Source data are provided as a Source Data file.

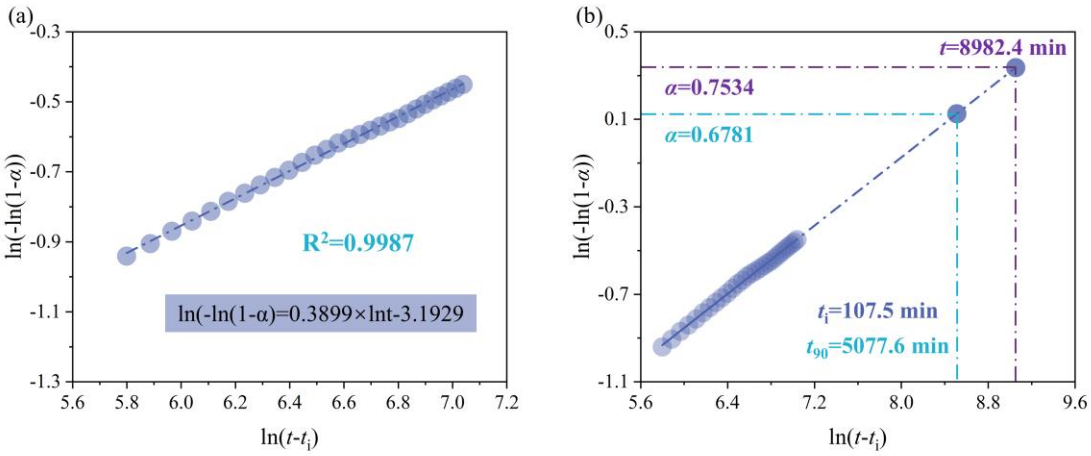

Supplementary Fig. 16. Avrami model fitting and extrapolation of CO_2@Water clathrate growth in SDS solution. (a) Avrami model of the final stage (437.5 min-1247.5 min) of CO_2@Water clathrate growth in SDS solution. **(b)** Duration and t_{90} for CO_2 capture capacity in SDS solution up to 109.4 v/v inferred from Avrami fit. (α represents the water to clathrate conversion, and α=0.7534 corresponds to the CO_2 storage capacity of 109.4 v/v; i represents induction time of CO_2@Water clathrate; α=0.6781=90%·$\alpha_{\text{total water to clathrate conversion}}$). Source data are provided as a Source Data file.

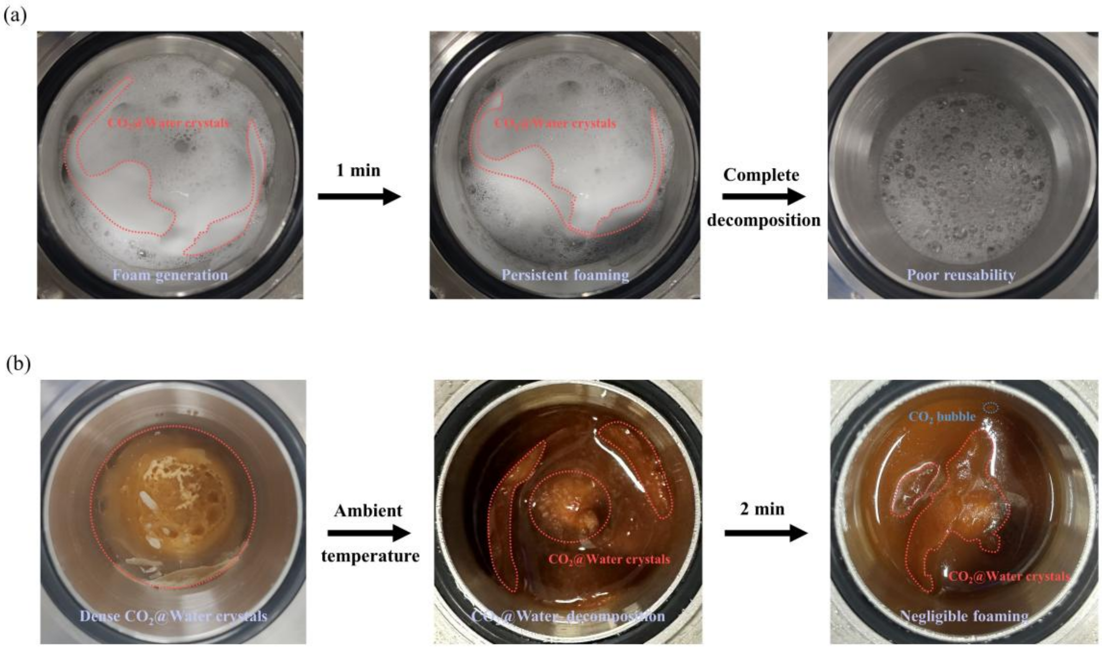

Supplementary Fig. 17. Comparison of foam generation during CO_2@Water clathrate decomposition in different systems. (a) SDS solution; **(b)** MFNs dispersion.

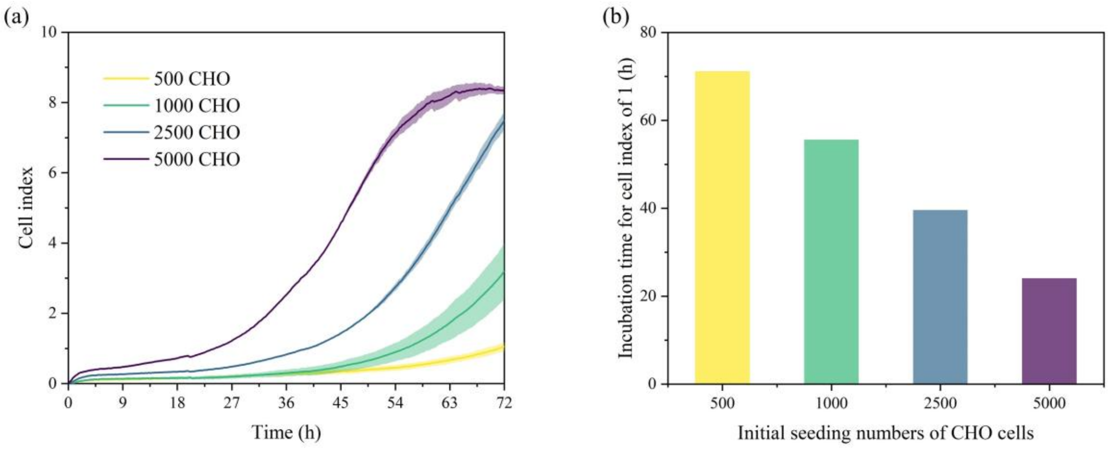

Supplementary Fig. 18. Determination of the optimal initial CHO seeding number. (a) The CHO cell growth curves measured by the RTCA system at different seeding numbers during the 72 h incubation. The error bars are determined from the standard deviation of three independent measurements. **(b)** Incubation time for cell index of ~1 at different seeding numbers. Source data are provided as a Source Data file.

3 Supplementary Tables

Supplementary Table 1. CO_2 capture performances and associated capture/regeneration conditions of representative crystalline precursors.

Crystalline precursor	CO_2 weight percent (%)	CO_2 capture condition	CO_2 release condition	Ref.
Hydroquinone	8.9	298 K, 20 bar	353K	[9]
HNIW (nitramine)	3.9	293 K, 160 bar	433K	[10]
P-tert-butylcalix[4]arene	7.0	298 K, 35 bar	High temperature	[11]
Azacalix[4]arene tetramethyl ether	2.4	195 K, 1 bar	-	[12]
Tetra-n-butylammonium bromide	7.5	282.65 K, 10.80 bar	Ambient condition	[13]
($Fe(bipy\text{-}NH_2)_3^{2+}$, bipy-$NH_2$ = 4,4′-diamino-2,2′-bipyridine)	21.6	Ambient condition	423 K	[14]
Gua_2SO_4	17.0	298 K, 0.52 bar	Ambient condition	[15]
Water	22.7	298-275 K, 40 bar	Ambient condition	This work

Supplementary Table 2. Comprehensive comparison of the advantages, regeneration temperatures, adaptation conditions and environmental impacts of several representative CO_2 capture methods.

Method	Advantages	Regeneration temperature	Environmental impacts	Adaptation conditions	Ref.
Liquid amine absorption	Capture efficiency, selectivity, and device compatibility	>100 ℃	Corrosive equipment, generation of degradation products	High CO_2 concentration	16
Porous material adsorption	Flexibility and recyclability	Spent zeolites: 250-300 ℃ Activated carbon: 70-200 ℃	No solvent contamination, recyclable materials	Low CO_2 concentration	17, 18
Carbonate crystallization	Capture efficiency and stable product	$CaCO_3$: >700 ℃	Strong corrosiveness of alkaline solutions	High CO_2 concentration	19
Hydrogen-bonded water cage	Capture capacity, insensitivity to impurities in industrial gases	Ambient (25°C)	Water as a crystallization precursor, green reaction	High CO_2 concentration	20

Supplementary Table 3. Element content in EDS of MFNs sample.

Element	Weight/%	Atom/%
C	18.16	31.10
O	42.20	54.25
S	0.23	0.15
Fe	39.40	14.51
Total	100.00	100.01

Supplementary Table 4. Comparison of costs and CO_2 capture capacity between MFNs and SDS.

Material	Reagent	Purity	Reagent cost* (\$/g)	Cost (\$/g)	CO_2 capture capacity (v/v)	t_{90} (min)
SDS	-	97%	0.0118	0.0118	79.0 ± 6.2	820.4 ± 98.3
MFNs	L-Met	98%	0.0188	0.0602	117.5 ± 2.1	352.9 ± 33.9
	$FeCl_3 \cdot 6H_2O$	90%	0.0033			
	$FeSO_4 \cdot 7H_2O$	90%	0.0035			
	NaOH	95%	0.0032			

Note: *The reagent cost comes from Shanghai Macklin Biochemical Technology Co., Ltd.

Supplementary Table 5. Adsorption enthalpy change (ΔH_{ads}) for typical CO_2 absorbents.

**Absorbent**	**ΔH_{ads} (kJ/mol)**	**Ref.**	**Absorbent**	**ΔH_{ads} (kJ/mol)**	**Ref.**
CaO	178	[21]	GBIG	151.5	[22]
Li_4SiO_4	142	[23]	Na_2CO_3	132.59	[24]
MgO	97.2	[23]	MEA	85.6	[25]
IL (amine-functionalized)	80	[26]	CO_2@Water clathrate	57.1	[27]
Activated carbon	43.96	[28]	MOF	47	[29]

Supplementary Table 6. CO_2 capture capacity of reported materials and operating conditions including concentration, temperature and pressure, stirring rate for CO_2@Water crystallization.

Promoter	Concentration (wt %)	Condition	Stirring (rpm)	Gas capture capacity (v/v)	Ref.
SDS	0.15	277.2 K, 35 bar	400	37.3	[6]
THF/Met	3.89	275.2 K, 34 bar	300	51.0	[30]
MWCNT-HB	0.8	273.7 K, 35 bar	-	72.8	[31]
1,3-dioxolane	19.5	280.7 K, 36 bar	150	63.1	[32]
Cu-Al LDH	1	275.2 K, 30 bar	200	20.8	[33]
S-H Graphenes	1	274.0 K, 31 bar	-	79.1	[34]
TBAB	32.08	282.2 K, 32 bar	-	8.7	[35]
THF	19.08	274.2 K, 30 bar	-	6.2	[36]
[BMIM][$FeCl_4$]	4.5	274.2 K, 50 bar	400	95.9	[37]
SGO	0.025	275.2 K, 37 bar	300	76.3	[38]
Coconut Fibers	3	274.2 K, 50 bar	150	80.5	[39]
ASA	0.1	275.0 K, 35 bar	-	99.1	[40]
Ag	0.0005	273.7 K, 32 bar	-	52.3	[38]
ZnO	0.1	274.0 K, 22 bar	500	85.9	[41]
VMNs	0.1	274.2 K, 34 bar	350	29.6	[42]
MFNs	0.5	275.2 K, 40 bar	100	118.7	This work

Note: THF: tetrahydrofuran; MWCNT-HB: modified herringbone carbon nanotube; Cu-Al LDH: Cu-Al layered double hydroxide; S-H graphenes: super-hydrophobic fluorinated graphenes; TBAB: Tetrabutylammonium bromide; SGO: graphene oxide with sulfonate groups ($-SO_3^-$); ASA: anionic surfactant based on aconitic acid; VMNs: vermiculite nanoflakes.

Supplementary Table 7. Comparison of CO_2 capture capacity and operating conditions of this scheme with typical porous materials and absorbents.

Sample	CO_2 weight percent (%)	Conditions		Ref.
		Temperature (K)	Pressure (bar)	
Activated Carbon	23.89	303	1	43
Mesoporous Carbon	14.08	298	1	44
BILP-5	8.70	298	1	45
BILP-5	12.80	273	1	45
CPC-550	25.60	298	1	45
HKUST-1	18.31	298	1	46
MOF-5	8.51	298	1	47
Mg-MOF-74	31.43	293	1	48
MIL-101	4.20	319	1	49
MCM-41	7.48	303	4	50
10GO-ZIF	9.63	273	1	51
IL-[BMIM][BF4]/CuBTC	19.64	298	35	52
COF-1	10.00	273	1	53
COF-1	23.00	298	55	54
COF-6	31.00	298	44	54
SBA-15-TA	7.04	273	1	55
Fe-POP-1	18.92	273	1	56
ZIF-8	2.95	298	1	57
MXene	8.90	298	1	58
Zeolite	16.72	298	20	59
N-F Clay	22.00	298	43.5	60
NaFh	11.51	298	33.7	60
MXene	2.65	298	10	61
MXene	25.48	298	40	62
Silica	20.24	298	30	63
COF-709	20.68	298	1	64
BCK-CTF-Gly	19.68	273	1	65
Zr-MOF	12.6	273	1	66
CO_2@Water clathrate	22.7	275	40	This work

Note: BILP: benzimidazole-linked polymer; CPC: porous N-doped carbon; MOF: metal–organic framework; HKUST-1: a representative MOF; MIL-101: a chromium-based MOF; MCM-41: a

functionalized ordered mesoporous silica; 10GO-ZIF: a composite of graphene oxide (GO) and zeolitic imidazolate framework (ZIF); COF: covalent organic framework; SBA-15-TA: N-propyl diethylenetriamine modified mesoporous silica; Fe-POP-1: iron containing porous organic polymer; MXene: 2D early transition metal carbides and/or nitrides; N-F Clay: nickel-fluorohectorite clay; NaFh: sodium fluorohectorite. BCK-CTF-Gly: A glycine-functionalized CTF.

Supplementary References

1. Shi C, *et al.* Pyrolytic aerogels with tunable surface groups for efficient methane solidification storage via gas hydrates. *Fuel* **331**, 125716 (2023).

2. Liu H, *et al.* Clay nanoflakes and organic molecules synergistically promoting CO_2 hydrate formation. *Journal of Colloid and Interface Science* **641**, 812-819 (2023).

3. Veluswamy HP, *et al.* An innovative approach to enhance methane hydrate formation kinetics with leucine for energy storage application. *Applied Energy* **188**, 190-199 (2017).

4. Falenty A, *et al.* Kinetics of CO_2 Hydrate Formation from Water Frost at Low Temperatures: Experimental Results and Theoretical Model. *The Journal of Physical Chemistry C* **115**, 4022-4032 (2011).

5. Susilo R, *et al.* Methane conversion rate into structure H hydrate crystals from ice. *AIChE Journal* **53**, 2451-2460 (2007).

6. Liu XJ, *et al.* Comparison of SDS and L-Methionine in promoting CO_2 hydrate kinetics: Implication for hydrate-based CO_2 storage. *Chemical Engineering Journal* **438**, (2022).

7. Dongre HJ, *et al.* Carbon Dioxide Hydrate Growth Dynamics and Crystallography in Pure and Saline Water. *Crystal Growth & Design* **20**, 7129-7140 (2020).

8. Zhao Y, *et al.* Magnetically Recyclable $-SO_3^-$-Coated Nanoparticles Promote Gas Storage via Forming Hydrates. *ACS Applied Materials & Interfaces* **14**, 33141-33150 (2022).

9. Torré J-P, *et al.* CO_2–Hydroquinone Clathrate: Synthesis, Purification, Characterization and Crystal Structure. *Crystal Growth & Design* **16**, 5330-5338 (2016).

10. Saint Martin S, *et al.* Host–Guest Inclusion Compound from Nitramine Crystals Exposed to Condensed Carbon Dioxide. *Chemistry – A European Journal* **16**, 13473-13478 (2010).

11. Thallapally PK, *et al.* Gas-induced transformation and expansion of a non-porous organic solid. *Nature Materials* **7**, 146-150 (2008).

12. Tsue H, *et al.* Crystallographic analysis of CO_2 sorption state in seemingly nonporous molecular crystal of azacalix 4 arene tetramethyl ether exhibiting highly selective CO_2 uptake. *Crystengcomm* **14**, 1021-1026 (2012).

13. Muromachi S, *et al.* Guest-induced symmetry lowering of an ionic clathrate material for carbon capture. *Chemical Communications* **50**, 11476-11479 (2014).

14. Luo YH, *et al.* Binding CO_2 from Air by a Bulky Organometallic Cation Containing Primary Amines. *Acs Applied Materials & Interfaces* **10**, 9495-9502 (2018).

15. Xiang Z, *et al.* Synthesis of stable single-crystalline carbon dioxide clathrate powder by pressure swing crystallization. *Cell Reports Physical Science* **4**, (2023).

16. Zhang X, *et al.* Amine-based CO_2 capture aided by acid-basic bifunctional catalyst: Advancement of amine regeneration using metal modified MCM-41. *Chemical Engineering Journal* **383**, 123077 (2020).

17. Singh G, *et al.* Emerging trends in porous materials for CO_2 capture and conversion. *Chemical Society Reviews* **49**, 4360-4404 (2020).

18. Ozkan M, *et al.* Progress in carbon dioxide capture materials for deep decarbonization. *Chem* **8**, 141-173 (2022).

19. Sanz-Pérez ES, *et al.* Direct Capture of CO_2 from Ambient Air. *Chemical Reviews* **116**, 11840-11876 (2016).

20. Wang P, *et al.* Hydrate Technologies for CO_2 Capture and Sequestration: Status and Perspectives. *Chemical Reviews* **124**, 10363-10385 (2024).

21. Sun J, *et al.* Evaluation of Thermochemical Energy Storage Performance of Fe-/Mn-Doped, Zr-Stabilized, CaO-Based Composites under Different Thermal Energy Storage Modes. *ACS Applied Energy Materials* **5**, 4903-4915 (2022).

22. Williams NJ, *et al.* CO_2 Capture via Crystalline Hydrogen-Bonded Bicarbonate Dimers. *Chem* **5**, 719-730 (2019).

23. Ding J, *et al.* Enhanced CO_2 adsorption of MgO with alkali metal nitrates and carbonates. *Applied Energy* **263**, 114681 (2020).

24. Kazemi H, *et al.* Optimization of CO_2 Capture Process Using Dry Sodium-Based Sorbents. (2020).

25. Galindo P, *et al.* Experimental research on the performance of CO_2-loaded solutions of MEA and DEA at regeneration conditions. *Fuel* **101**, 2-8 (2012).

26. Gurkan BE, *et al.* Equimolar CO_2 Absorption by Anion-Functionalized Ionic Liquids. *Journal of the American Chemical Society* **132**, 2116-2117 (2010).

27. Qiu N, *et al.* Grand Canonical Monte Carlo Simulations on Phase Equilibria of Methane, Carbon Dioxide, and Their Mixture Hydrates. *The Journal of Physical Chemistry B* **122**, 9724-9737 (2018).

28. Guo Y, *et al.* Porous activated carbons derived from waste sugarcane bagasse for CO_2

adsorption. *Chemical Engineering Journal* **381**, 122736 (2020).

29. Caskey SR*, et al.* Dramatic Tuning of Carbon Dioxide Uptake via Metal Substitution in a Coordination Polymer with Cylindrical Pores. *Journal of the American Chemical Society* **130**, 10870-10871 (2008).

30. Liu XJ*, et al.* Coupling Amino Acid with THF for the Synergistic Promotion of CO_2 Hydrate Micro Kinetics: Implication for Hydrate-Based CO_2 Sequestration. *Acs Sustainable Chemistry & Engineering* **11**, 6057-6069 (2023).

31. Liu N*, et al.* Investigating the effects of MWCNT-HB on gas storage performance of CO_2 hydrate. *Fuel* **316**, (2022).

32. Yao YX*, et al.* Evaluation of 1,3-dioxolane in promoting CO_2 hydrate kinetics and its significance in hydrate-based CO_2 sequestration. *Chemical Engineering Journal* **451**, (2023).

33. Ansari AA*, et al.* Synthesis of Cu-Al LDH nanofluid and effectiveness as a promoter for CO_2 hydrate formation. *Chemical Engineering Journal* **435**, (2022).

34. Deng ZX*, et al.* High storage capacity and high formation rate of carbon dioxide hydrates via super-hydrophobic fluorinated graphenes. *Energy* **264**, (2023).

35. Li XY*, et al.* Investigation of Tetra-n-Butyl Ammonium Bromide Semiclathrate Hydrate-Based CO_2 Capture by Kinetic and In Situ Raman Spectroscopy Measurement. *Industrial & Engineering Chemistry Research*, (2023).

36. Veluswamy HP*, et al.* Effect of guest gas on the mixed tetrahydrofuran hydrate kinetics in a quiescent system. *Applied Energy* **207**, 573-583 (2017).

37. Liu FP*, et al.* Iron-based ionic liquid (BMIM $FeCl_4$) as a promoter of CO_2 hydrate nucleation and growth. *Energy* **214**, (2021).

38. He Y*, et al.* Hydrate-based CO_2 capture: kinetic improvement via graphene-carried $-SO_3^-$ and Ag nanoparticles. *Journal of Materials Chemistry A* **6**, 22619-22625 (2018).

39. Trivedi V*, et al.* Enhancing CO_2 hydrate formation: Effect of coconut fibers on nucleation kinetics of CO_2 hydrates. *Journal of Crystal Growth* **549**, (2020).

40. Sadeh E*, et al.* Energy-efficient storage of methane and carbon dioxide capture in the form of clathrate hydrates using a novel non-foaming surfactant: An experimental and computational investigation. *Energy Conversion and Management* **293**, (2023).

41. Mohammadi M*, et al.* Experimental study and thermodynamic modeling of CO_2 gas

hydrate formation in presence of zinc oxide nanoparticles. *Journal of Chemical Thermodynamics* **96**, 24-33 (2016).

42. Liu HQ*, et al.* Clay nanoflakes and organic molecules synergistically promoting CO_2 hydrate formation. *Journal of Colloid and Interface Science* **641**, 812-819 (2023).

43. Yaumi AL*, et al.* Melamine-nitrogenated mesoporous activated carbon derived from rice husk for carbon dioxide adsorption in fixed-bed. *Energy* **155**, 46-55 (2018).

44. Wei J*, et al.* A Controllable Synthesis of Rich Nitrogen-Doped Ordered Mesoporous Carbon for CO_2 Capture and Supercapacitors. *Advanced Functional Materials* **23**, 2322-2328 (2013).

45. Ashourirad B*, et al.* Exceptional Gas Adsorption Properties by Nitrogen-Doped Porous Carbons Derived from Benzimidazole-Linked Polymers. *Chemistry of Materials* **27**, 1349-1358 (2015).

46. Chen C*, et al.* Synthesis of Hierarchically Structured Hybrid Materials by Controlled Self-Assembly of Metal Organic Framework with Mesoporous Silica for CO_2 Adsorption. *Acs Applied Materials & Interfaces* **9**, 23060-23071 (2017).

47. Zhao ZX*, et al.* Adsorption and Diffusion of Carbon Dioxide on Metal-Organic Framework (MOF-5). *Industrial & Engineering Chemistry Research* **48**, 10015-10020 (2009).

48. Chakraborty A*, et al.* Mg-MOF-74@SBA-15 hybrids: Synthesis, characterization, and adsorption properties. *Apl Materials* **2**, (2014).

49. Chowdhury P*, et al.* Gas Adsorption Properties of the Chromium-Based Metal Organic Framework MIL-101. *Journal of Physical Chemistry C* **113**, 6616-6621 (2009).

50. Tari NE*, et al.* One pot microwave synthesis of MCM-41/Cu based MOF composite with improved CO_2 adsorption and selectivity. *Microporous and Mesoporous Materials* **231**, 154-162 (2016).

51. Chen BL*, et al.* Controlled *in situ* synthesis of graphene oxide/zeolitic imidazolate framework composites with enhanced CO_2 uptake capacity. *Rsc Advances* **5**, 30464-30471 (2015).

52. Sezginel KB*, et al.* Tuning the Gas Separation Performance of CuBTC by Ionic Liquid Incorporation. *Langmuir* **32**, 1139-1147 (2016).

53. Olajire AA. Recent advances in the synthesis of covalent organic frameworks for CO_2 capture. *Journal of CO_2 Utilization* **17**, 137-161 (2017).

54. Furukawa H, *et al.* Storage of Hydrogen, Methane, and Carbon Dioxide in Highly Porous Covalent Organic Frameworks for Clean Energy Applications. *Journal of the American Chemical Society* **131**, 8875-8883 (2009).

55. Zelenák V, *et al.* Carbon dioxide adsorption over amine modified silica: Effect of amine basicity and entropy factor on isosteric heats of adsorption. *Chemical Engineering Journal* **348**, 327-337 (2018).

56. Modak A, *et al.* Porphyrin based porous organic polymers: novel synthetic strategy and exceptionally high CO_2 adsorption capacity. *Chemical Communications* **48**, 248-250 (2012).

57. Gao F, *et al.* Dynamic hydrophobic hindrance effect of zeolite@zeolitic imidazolate framework composites for CO_2 capture in the presence of water. *Journal of Materials Chemistry A* **3**, 8091-8097 (2015).

58. Arifutzzaman A, *et al.* MXene based activated carbon novel nano-sandwich for efficient CO_2 adsorption in fixed-bed column. *Journal of CO_2 Utilization* **68**, (2023).

59. Garshasbi V, *et al.* Equilibrium CO_2 adsorption on zeolite 13X prepared from natural clays. *Applied Surface Science* **393**, 225-233 (2017).

60. Cavalcanti LP, *et al.* A nano-silicate material with exceptional capacity for CO_2 capture and storage at room temperature. *Scientific Reports* **8**, (2018).

61. Petukhov DI, *et al.* MXene-based gas separation membranes with sorption type selectivity. *Journal of Membrane Science* **621**, (2021).

62. Wang BX, *et al.* Carbon dioxide adsorption of two-dimensional carbide MXenes. *Journal of Advanced Ceramics* **7**, 237-245 (2018).

63. Knöfel C, *et al.* Functionalised micro-/mesoporous silica for the adsorption of carbon dioxide. *Microporous and Mesoporous Materials* **99**, 79-85 (2007).

64. Li H, *et al.* Bonding of Polyethylenimine in Covalent Organic Frameworks for CO_2 Capture from Air. *Journal of the American Chemical Society* **146**, 35486-35492 (2024).

65. Dong B, *et al.* Post synthesis of a glycine-functionalized covalent triazine framework with excellent CO_2 capture performance. *Microporous and Mesoporous Materials* **306**, 110475 (2020).

66. Wang B, *et al.* Metal-Modified Zr-MOFs with AIE Ligands for Boosting CO_2 Adsorption and Photoreduction. *Advanced Materials* **n/a**, 2407154 (2025).
