# Supplementary material for: Organic magnetic nanoparticles catalyze CO2 capture in hydrogen-bonded nanocages via water-driven crystallization
Source: Nat Commun. 2025 Apr 18;16:3702. doi: 10.1038/s41467-025-58734-1 (PMC12008246; doi:10.1038/s41467-025-58734-1)
Supplement: Supplementary file 3 — Description of Additional Supplementary Files [file 41467_2025_58734_MOESM3_ESM.pdf]

Description of Additional Supplementary Files

Supplementary Movie 1: MD simulations for CO_2 capture process in hydrogen-bonded water cages within 2000 ns.

Supplementary Movie 2: Structural evolution of hydrogen-bonded water cage within 0.5 nm surrounding a CO_2 molecule involved in CO_2@Water crystal nucleation within 2000 ns MD simulations.
